# The Importance of Species Name Synonyms in Literature Searches

**DOI:** 10.1371/journal.pone.0162648

**Published:** 2016-09-14

**Authors:** Gerald F. Guala

**Affiliations:** Core Science Analytics, Synthesis and Libraries Program, Core Science Systems Mission Area, U.S. Geological Survey, Reston, Virginia, United States of America; University of Innsbruck, AUSTRIA

## Abstract

The synonyms of biological species names are shown to be an important component in comprehensive searches of electronic scientific literature databases but they are not well leveraged within the major literature databases examined. For accepted or valid species names in the Integrated Taxonomic Information System (ITIS) which have synonyms in the system, and which are found in citations within PLoS, PMC, PubMed or Scopus, both the percentage of species for which citations will not be found if synonyms are not used, and the percentage increase in number of citations found by including synonyms are very often substantial. However, there is no correlation between the number of synonyms per species and the magnitude of the effect. Further, the number of citations found does not generally increase proportionally to the number of synonyms available. Users looking for literature on specific species across all of the resources investigated here are often missing large numbers of citations if they are not manually augmenting their searches with synonyms. Of course, missing citations can have serious consequences by effectively hiding critical information. Literature searches should include synonym relationships and a new web service in ITIS, with examples of how to apply it to this issue, was developed as a result of this study, and is here announced, to aide in this.

## Introduction

Latin binomials, the scientific names of biological species, are typically used as terms in searches for literature across the biological disciplines. However, because synonyms for those binomials often exist, the efficiency of individual binomials in guaranteeing retrieval of all relevant items can be greatly affected by the inclusion (or exclusion) of synonyms in the search. In biological nomenclature, synonyms are scientific names, other than the currently accepted one, that apply to an organism. The purpose of this study is to assess the importance of this effect in online searches of a popular cross-section of relevant scientific literature databases.

In this paper, I provide a repeatable and quantifiable snapshot of the effect of synonyms on searches in a very large commercial scientific literature index, Scopus [1], two related public indexes, PubMed [2] and PubMed Central [3] and the smaller but broader Public Library of Science (PLoS)[4]. Search queries were all generated and conducted using only online interfaces. The general idea was simply to search every accepted or valid species in the Integrated Taxonomic Information System ITIS [5] that had at least one synonym, against each of the aforementioned online databases and then run each of those searches again including all synonyms for each species, thus allowing a direct assessment of the effect of synonyms in the number of citations retrieved. Binomials were chosen as the units of comparison because of their unique combination of a specific rank (species) with a unique lexical structure consisting, in canonical form, of an ordered string of two paired tokens, the genus and specific epithet. This allows a much higher degree of comparability in results than one would get with the inclusion of monomials, while still retaining a much larger sample than would be gotten with trinomials alone. Trinomials, also suffer from a high level of variance in their presentation in the literature with respect to whether or not a rank indicator is employed in the string, what the abbreviation for that indicator is, and when not present, in the rank itself.

The Integrated Taxonomic Information System is an authoritative source for the currently used names of all biological taxa for many U.S. Federal agencies. While richest in North American taxa, it has a global scope and treats high profile groups such as vertebrates, several insect and plant groups, and prokaryotes at a global level. An aspect of ITIS critical for this study is that it contains a single classification. There are no ambiguities in the designation of names as accepted (plants) or valid (animals) versus as synonyms. At the time of the extraction of species lists for the study, 20 December 2015, there were 708,566 scientific names in ITIS. Of these 526,079 were species binomials and 397,243 were accepted or valid species names. Counting all synonyms, which can include names published at other ranks, 537,276 names were either valid or accepted species names or synonyms of those names. Species2000 and ITIS collaborate each year to compile the broader resource called Catalogue of Life (CoL) [6], however, unlike the CoL, ITIS retains all names ever used with unique, guaranteed stable and fully persistent identifiers called Taxonomic Serial Numbers or TSNs allowing long term reproducibility. While linkages may change over time with advancements in taxonomic knowledge, TSNs and their forever associated names never disappear, and all names are linked to an accepted/valid parent taxon in a complete hierarchy. Importantly, it must be recognized that TSNs are linked to the names themselves, not their usage. All accepted/valid species binomials in ITIS with at least one synonym (and all of their synonyms) were used in more than 1.6 million name string searches contained in more than 800,000 URLs across the four sources. The term “synonym” in this paper is used in a relatively broad sense to include all name strings linked directly to an accepted or valid name in ITIS. While orthographic variants and similar variations are sometimes included, informal designations that do not follow accepted Latin form are not.

Scopus is the largest commercial abstract and citation database of peer-reviewed literature: scientific journals, books and conference proceedings. It currently contains nearly 60 million items and covers disciplines very broadly with more than 40,000 books and 34,496 periodical titles [7]. PubMed and PubMed Central are Federal databases produced by the National Library of Medicine and the National Center for Biotechnology Information. PubMed comprises more than 25 million citations for biomedical literature from MEDLINE, life science journals, and online books. PubMed Central archives more than 3.7 million articles in full text from 5612 journals. The PLOS suite of journals includes original research from all disciplines within science and medicine. Currently, the PLoS index contains more than 167,000 articles, >94% of which include abstracts. There is significant overlap among these resources but each presents a unique view of the literature and serves different audiences. They were also chosen because they have high speed APIs that allow repeated large scale sets of queries.

Hopefully, this work will serve as a baseline for later studies looking at the same issues as this one but in hindsight after efforts have been made to address the problem, as well as an indication of the importance of synonyms in online searches.

## Materials and Methods

A set of paired query URLs for each resource [S1 File] (Plos, Scopus, PubMed and PMC) by kingdom [8] was generated from the new ITIS Solr web service [9]. These consisted of one query URL for each accepted or valid name in ITIS at the species rank with at least one synonym in ITIS and another URL for each accepted or valid name plus all of its linked synonyms in ITIS using exact phrase delimiters for each name string and an inclusive Boolean “OR” according to the specific requirements of each resource. The synonyms could include names at other ranks (e.g. subspecies and varieties) but not valid or accepted children. One set for each resource was generated to query all available fields. For PubMed an additional set was generated to query only the Title and Abstract, and for Scopus a set to query only the Abstract as well. Result reports for each resource were constrained to the smallest response deliverable in JSON that reported the number of citations obtained for the individual search being conducted. Actual text fields from the articles themselves were not downloaded other than in a test set of records for each resource to confirm search request fidelity to expected results. All URLs were batch submitted using GNU WGet version 1.16.3 [10] and required information was extracted from the JSON responses using simple javascript functions. Sample URL strings (minus API keys) are provided in the associated data for this paper.

Two species, *Anodonta anatina* and *A*. *cygnea*, had 303 and 285 synonyms respectively. Individual sets were generated for each of those species to query them separately for each source by individual synonym to investigate the contributions of individual synonyms and because their ensemble Boolean synonym strings exceeded the query string length limit for all sources. Due to differences in what individual services would accept, such as maximum query string length and different special characters, there was some variance in the actual number of names successfully queried per source. This amounted to less than 0.1% of names. However, a base set of 67,187 accepted or valid species names and their 139,207 synonyms were successfully queried for every source.

For all resources, a small subset of searches run through the web APIs were also run manually on the primary public graphical user interface for each resource to confirm that results were consistent between the API and public portals. No divergence was seen. Every effort was made to divide queries into subsets and run them at reduced rates if necessary to meet the access policies of individual resources. The NCBI resources (PubMed and PMC) are fully open. The standard free API key was used for PLoS and Elsevier generously provided a higher volume API key for Scopus when the limits of their free API key were reached.

## Results

Among all valid or accepted species names in ITIS, only those with synonyms (~17%) were used in this study, and among those, the number yielding any citations at all varied widely across kingdoms and sources. See supporting documentation. However, for those species that had synonyms, and citations in the literature, both the number of species affected by adding synonyms to the search string (See Table 1), and the number of citations returned (See Table 2), was generally substantial.

**Table 1:** Percent of Accepted or Valid Names with Synonyms and Citations for which Synonyms Added Citations.

	PLoS	PubMed	PMC	Scopus
Animalia	3.1	25.1	20.4	38.3
Archaea	44.0	89.6	92.0	98.0
Bacteria	30.9	80.3	81.2	87.7
Chromista	4.6	25.4	30.3	41.5
Fungi	4.9	29.3	41.3	41.3
Plantae	5.2	29.7	26.9	40.6
Protozoa	4.8	30.0	25.0	48.0

**Table 2:** Percent Increase in Number of Citations Found Due to Synonyms.

	PLoS	PubMed	PMC	Scopus
Animalia	8.6	16.5	7.4	19.0
Archaea	25.2	40.1	32.7	72.4
Bacteria	18.8	18.5	25.1	31.8
Chromista	22.7	27.1	24.0	38.1
Fungi	0.6	0.7	0.8	1.2
Plantae	11.3	19.7	11.6	17.0
Protozoa	22.2	11.4	12.4	32.7

The effect of individual synonyms was investigated in the two mussel species previously mentioned (*Anodonta anatina* and *A*. *cygnea*) as well as in all 13,487 accepted species with more than one synonym that yielded citations in the PubMed automated search. The number of citations found did not generally increase proportionally to the number of synonyms available. For example, in PubMed (See S3 File for the full set of relevant results) although 16 or more synonyms were searched for 355 species and the number of synonyms searched per species in that group went as high as 127, no more than 16 synonyms actually yielded results for any single species search. Even in the two *Anodonta* species searched individually, while a few synonyms sometimes yielded results individually, in their richest set (Scopus), no more than one synonym was ever needed with the valid name to yield the same number of results as in searching that valid name and all other synonyms. Because multiple synonyms can be present in the same paper, one can’t assume that the results of independent synonyms are independent. The ensemble effect of synonym number per species with respect to both the raw number of citations returned and with the difference between searches with, and without, synonyms was determined by computing the Pearson product-moment correlation coefficient on the Scopus data which was the richest set. There was no significant correlation between the number of synonyms that a species had with either the number of raw citations returned for that species, or the increase, if any, in the number of citations returned with synonyms versus without. The coefficient was 0.04 in both cases.

In Scopus there are rarely synonyms in a keyword field, and in PubMed and PMC this is also the case. PubMed and PMC also have an added redirection in search to use MeSH terms when a species is not found among the citations but found in MeSH. This is also a rare and serendipitous case because MeSH does not maintain species level synonymy. In runs of all species against both resources querying only the abstracts in the case of Scopus and the title and abstract in the case of PubMed, of course the raw numbers of citations returned were greatly diminished. In PubMed the percentage of species for which citations were found and synonyms made a difference in the number of citations returned remained within 1% of the values obtained for the full record search in all kingdoms. In Scopus, there was wider variance with a reduction of up to 14% (in the case of Animalia) but an increase of 4.7% Chromista. Further heuristic investigation of the Scopus data suggested that the effect was primarily due to Scopus having richer records in many cases with full titles of articles in the references (and thus having searchable species names in those titles) and with species names in the full text of the many articles in Scopus that have it, not just the abstract.

## Conclusions

All of the major resources examined in this study are heavily used and support millions of visits a month [11]. Of course, only some fraction of those visits are for searches in which the user wants literature about a given species or set of species. However, it seems to be a reasonable assumption that there is a high interest if only because ITIS species names appear in at least 8 million citation records returned for only the searches of species with their synonyms in Scopus alone. Further, ITIS is neither taxonomically complete, nor does it include all possible synonyms where it is complete. And, of course, there can be other taxonomic views. However, with any complete taxonomic view, the relationships may change but the totality of names will not. The exact search goals of each user, and optimization of metrics for searches are of course a much broader question, but because a scientist can’t evaluate what isn’t known to exist, we must assume for the purposes of this paper that more results are better in this case. And it is clear that the inclusion of synonyms increases results. As shown here with both the minimal effect of large numbers of synonyms [S3 File], and the low correlation of number of synonyms to number of citations returned across all taxa and sources [S2 File], the relative value of the large investments needed to achieve exhaustive synonymies needs further justification if the goal is for efficient literature search.

The value of synonyms in general, especially those in common use at one time, is clear. At the individual species level, a given source in this study may or may not have had citations for the accepted name of a species or its synonyms, but across all sources and kingdoms, there was always at least a minimal effect of adding synonyms to the search. And sometimes, such as in Archaea, nearly all searches for names that had synonyms were incomplete without those synonyms. Strangely with the NCBI databases, there is actually a limited set of synonymy available within the separate NCBI taxonomy database, but it is not automatically integrated into their general search like MeSH is. One could use it separately and transfer the results manually, or build a service to use it in an integrated way, but I found none available at this point. The bottom line is that users looking for literature on specific species across all of the resources investigated here are often missing large numbers of citations if they are not manually augmenting their searches with synonyms. This can have serious consequences. The range of a species known by different names in different regional treatments will erroneously appear restricted. A physician treating a pathogen known more commonly by another name will be blind to the bulk of potentially life-saving literature.

Stemming directly from the results obtained from the preliminary work associated with this study, ITIS has implemented within a high speed Solr web service [9], the ability to retrieve a list of synonyms for the accepted or valid name, associated with any synonym, as well as for the accepted or valid names themselves. Output is available in JSON, JSONP, Serialized PHP or XML. This allows their efficient use in dynamic search string expansion for virtually any web application, or as a way to add synonyms to any existing database. A simple example application which takes a user entered species name and constructs an appropriately formatted search string of the accepted or valid name of the species along with all of its synonyms (as used in this study) and then sends that to the chosen resource for query is provided on the ITIS website [12]. The application also exploits this capability for commercial search engines which no longer allow the kind of large scale automated interrogation used in this study (Google, Google Scholar and Bing). Any user trying one of these examples will see that the conclusions reached here also currently extend very similarly to those resources as well.

## Supporting Information

S1 File URL Formats for All Sources. (TXT)

S2 File Full Results for Scopus with PPMCC Data. (CSV)

S3 File Results for PubMed species with more than one synonym yielding citations. (CSV)
